# SSDoH in Latinxs: Factors of Influence

**DOI:** 10.1093/geroni/igab046.305

**Published:** 2021-12-17

**Authors:** David Marquez

**Affiliations:** University of Illinois at Chicago & Rush University, University of Illinois at Chicago, Illinois, United States

## Abstract

Research with Latinxs/os/as regarding Alzheimer’s disease and related dementias (ADRD) is lacking. This is staggering because among Latinxs in the United States, the number diagnosed with ADRD is expected to grow by more than 800% from 2012 to 2060 (Wu et al., 2016). Older Latinxs have a high risk and prevalence of ADRD - partially attributed to their longer life spans and the presence of adverse risk factors such as metabolic syndrome, type 2 diabetes mellitus, and other cardiovascular conditions (Chin et al., 2011). What is often missing in the discussion is the role of social and structural determinants of health (SSDOH) in this population. Overall Latinxs have low levels of formal education, work in physically demanding jobs, and experience immigration stress. How these and other SSDoH influence Latinxs will be discussed; as well as potential resilience factors like familial relationships, and religiosity or spirituality.

